# Sonophore enables autonomous observation of micronekton communities in the ocean twilight zone

**DOI:** 10.1038/s41598-026-41581-5

**Published:** 2026-03-02

**Authors:** Ryan A. Downie, Peter Jansen, Gavin J. Macaulay, Yoann Ladroit, Heidi R. Pethybridge, Simon J. Nicol, Campbell R. Davies

**Affiliations:** 1 CSIRO Environment, Hobart, Australia; 2 CSIRO National Collections and Marine Infrastructure, Hobart, Australia; 3 Aqualyd Ltd, Wakefield, New Zealand; 4 Kongsberg Discovery, Horten, Norway; 5 Oceanic Fisheries Programme, The Pacific Community, BP D5 , 98848 Noumea, New Caledonia; 6 Institute for Applied Ecology, Centre for Conservation Ecology and Genomics, University of Canberra, Bruce, ACT 2617 Australia

**Keywords:** Twilight zone, Micronekton, Biogeochemical cycles, Pelagic ecosystem, Climate change, Marine acoustics, Environmental monitoring, Ecology, Ecology, Ocean sciences

## Abstract

The future productivity of pelagic ecosystems and fisheries globally remains uncertain due to a lack of data on mid-trophic mesopelagic micronekton communities. Here, we demonstrate that integrating readily available autonomous profiling floats with autonomous echosounders enables vertically resolved abundance estimates of mesopelagic micronekton communities, a platform we are naming the “Sonophore”. This successful demonstration is a first step towards addressing critical data gaps identified by fisheries management, earth system and ecosystem modelling communities. With planned engineering enhancements, this platform offers a scalable solution for rapid, cost-effective, year-round monitoring of the planet’s largest vertebrate (but deeply uncertain) biomass. The platform is specifically designed for long-term monitoring of remote and spatially extensive oceanic habitats of the global ocean, without the need for large, expensive research vessels.

## Introduction

The oceans mesopelagic zone, spanning depths of 200 to 1000 m, is dominated by mesopelagic micronekton communities including small fish, crustaceans, squid and gelatinous zooplankton. These organisms play fundamental roles in global biogeochemical cycles and climate regulation through their extensive diel vertical migrations and high abundance^1–4^. By actively transporting carbon, nutrients, and energy between surface and deep waters they contribute to the biological carbon pump and sustain pelagic predator populations globally, including commercially important tunas^5,6^. The efficiency and magnitude of these processes vary widely across oceanic regions, particularly between oligotrophic gyres and eutrophic upwelling regions, resulting in complex spatial patterns in ecosystem structure and function^7^.

Despite the biochemical and ecological importance of micronekton communities, global biomass estimates remain highly uncertain, with acoustic estimates varying between 1.8 and 16 Gt, with studies calling for coordinated ground-truthing studies and the use of new technologies to reduce uncertainty^8–10^. The distribution, abundance, and community dynamics are difficult to monitor using conventional sampling tools because micronekton are widely distributed, fragile and because ship-based surveys are selective and costly^11^. Autonomous profiling acoustic and optical systems have been identified as tools that may reduce uncertainties in acoustically derived global biomass estimates of micronekton^1,10^. Acoustic observations from autonomous profiling platforms offer several benefits compared to surface-based platforms. They observe individuals at close range instead of integrating backscatter from large volumes of water, extend the use of high frequency acoustics to greater depths, and can remain in a region for a sustained period, enabling characterisation of seasonal and interannual variability in micronekton community dynamics.

Earth system and ecosystem models have until relatively recently, tended to exclude or oversimplify mid-trophic micronekton communities, which generates substantial uncertainties in projections of oceanic carbon sequestration, ecosystem production, and climate driven ecosystem change^12–14^. Autonomous profiling echosounders can help address these gaps directly by quantifying community composition and diel vertical migration dynamics, measurements that are essential for constraining biological carbon-pump processes and discriminating contrasting ecosystem regimes. For example, eutrophic systems typically have diverse, abundant micronekton communities dominated by krill and small pelagic fish, while oligotrophic systems support fewer, deeper-migrating species^15–17^. Resolving these patterns in situ is critical for validating spatially explicit operational ecosystem models such as SEAPODYM and for stand-alone monitoring of these key mid trophic functional groups^18^.

**Fig. 1:**
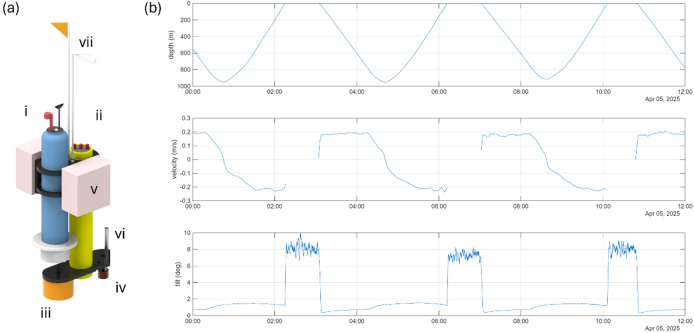
Schematic diagram of the Sonophore (**a**) and a twelve-hour subset of Sonophore dive data from 5th April 2025 displaying data, platform depth (m), velocity (m/s) and tilt (degrees) (**b**). (**a**) Sonophore components i: MRV Alto with RBR CTD; ii: Kongsberg WBAT; iii: Single beam 38 kHz transducer; iv: Split-beam 120 kHz transducer; v: Syntactic foam flotation; vi: Lowell Instruments Orientation and Acceleration (MAT1)  sensor; vii: Flag and recovery loop.

Technological advances in autonomous ocean observing platforms offer new opportunities to address longstanding data gaps at the spatiotemporal scale required to improve our understanding of the role of this important functional group in pelagic ecosystems. This paper presents a proof of concept that demonstrates how standard autonomous floats can be combined with echosounders to create a cost-effective platform for monitoring micronekton distribution and abundance at relevant spatiotemporal scales (Fig. 1a).

## Results

Two commercially available self-contained, broadband echosounders combined with off-the-shelf profiling floats were deployed and recovered from a commercial fishing vessel in waters off the Tasmanian coast in April 2025. During the 102-hour mission, profiling echosounders completed 24 unassisted dives from the surface to ~ 1000 m. On average floats drifted 3.1 km over a 24-hour period in an eastward direction, following surface currents that flowed in the same direction at ~ 0.2 m/s (https://oceancurrent.aodn.org.au/). Each dive took approximately 3 h, with the remaining time spent at the surface (about 30% of the deployment period). The platform profiled on average at 0.2 m/s and remained vertically stable while profiling with an average tilt of one degree (Fig. 1b). The echosounder generated approximately 20,000 pings on each dive and recorded the broadband backscattering cross section (TS dB re 1 m^2^) from an average of 6875 organisms per dive. The acoustic data quality was high with minimal noise and interference.

In its current configuration the echosounder can operate for approximately 360 h, equivalent to ~ 102 profiles from 1000 m to the surface. Profiling every 10 days, following standard Argo protocol, the Sonophore echosounder has the capacity operate for ~ 2.5 years compared to the MRV Alto float that has expected operational life of ~ 5 years or 500 profiles.

**Fig. 2:**
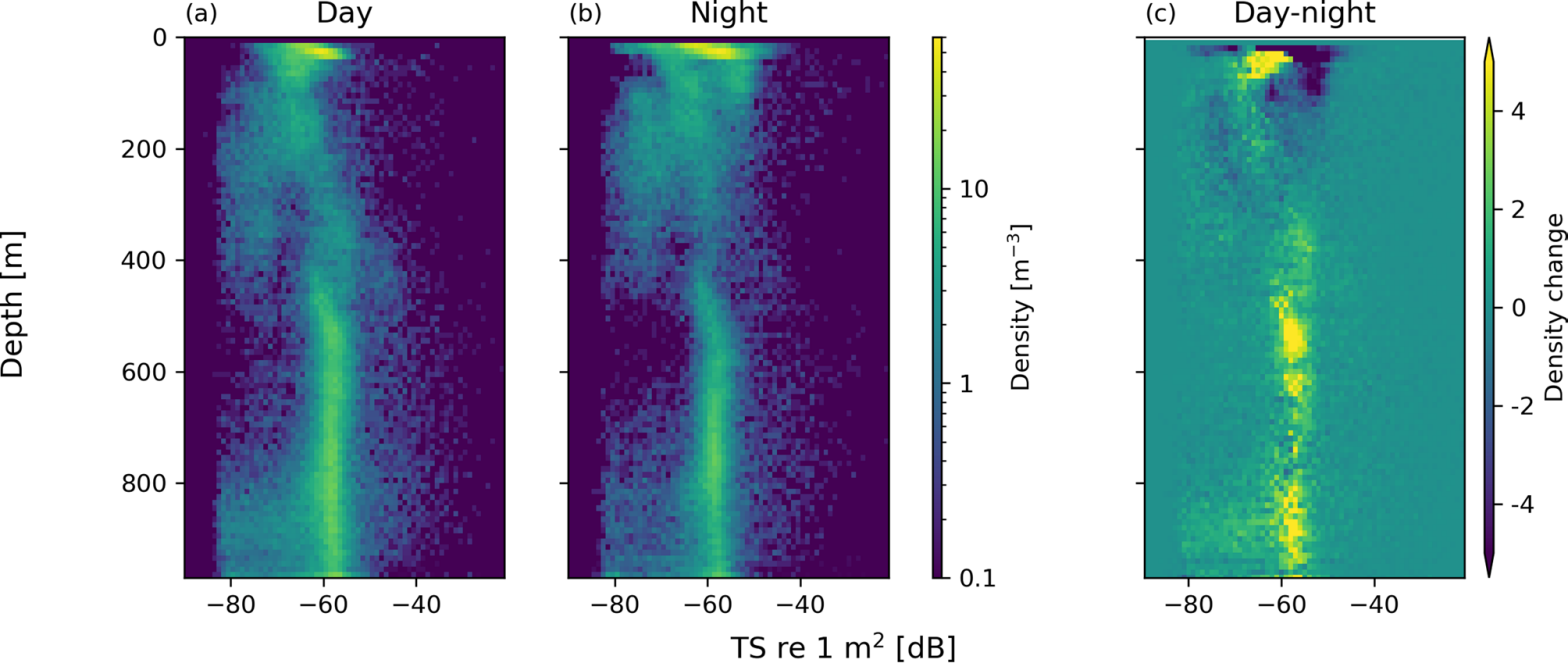
Depth-resolved distribution of organism volumetric density and backscatter strength (TS re 1m^2^ [dB] at 120 kHz) distribution during the day (**a**) and night (**b**), and the difference between day and night (**c**). Positive values in (**c**) indicate higher densities during the day and negative values higher densities during the night.

The acoustic dataset provided high-resolution information on the volumetric density (number of organisms per unit volume), vertical distribution, and an individuals backscattering cross section (i.e. target strength, TS) from which size can be inferred. No forms of organisms avoidance were readily detectible in the analysed acoustic records. Clear diel differences in volumetric density were observed between day and night (Fig. 2), consistent with diel vertical migration behaviour of mesopelagic organisms. During the day, volumetric densities were highest below 450 m, while at night organisms, particularly those with high TS (indicative of larger organisms), aggregated in the upper 100 m of the water column. Subtle variations in organism density between 100 and 450 m suggest additional behavioural changes influencing vertical positioning and acoustic detectability.

## Discussion

Global biomass estimates of mesopelagic micronekton currently vary by a factor of 9 (1.8–16 Gt) reflecting fundamental challenges in studying these remote and vast ecosystems acoustically^10,19,20^. This uncertainty largely stems from biases inherent in existing sampling methods and limited global samples. As a result, our understanding of how mesopelagic communities respond to variability in physical and ecological drivers remains limited but is undoubtably important^21–23^. This knowledge gap means that biogeochemical and ecosystem models treat mesopelagic micronekton crudely, while most Earth System models do not include them at all, constraining their predictive capacity and relevance for climate and fisheries management^24^.

This study provides a proof of concept that autonomous float-mounted echosounders can resolve micronekton distribution and abundance at high resolution for sustained periods of time. The ability of the float to profile independently at defined time intervals demonstrates that the Sonophore concept is a step towards a scalable, cost-effective platform capable of addressing longstanding knowledge gaps. In contrast to traditional ship-based surveys, moorings and gliders equipped with echosounders - which are constrained by high costs, restricted temporal or spatial coverage and time-consuming deployment management (gliders) - the Sonophore can, in principal, autonomously observe micronekton communities over sustained periods to characterise their dynamics. When sufficient data are acquired at relevant spatiotemporal scales, these data will allow for tighter constraints on the parameters governing both the biogeochemical mesopelagic migrant pump and the gravitational pumps, thereby improving estimates of carbon export in global biogeochemical models and ecosystem productivity^25,26^.

While the Sonophore represents a step forward in autonomous observations of open ocean micronekton communities, the current implementation is assembled from readily available equipment. Several limitations must be addressed for the concept to be fully operational: (1) coordination of profiling activities with echosounder operations; (2) reductions in echosounder power consumption and enhanced battery capacity to match a standard Argo floats profiling capacity (500 dives); (3) transceivers capable of running four transducers simultaneously to improve acoustic categorisation and inversion based size estiamtes; (4) onboard processing to produce data products suitable for transmission, i.e. summaries of an organism’s target strength information; (5) transmission of data summaries ashore via a satellite link. These limitations are technological in nature and can be addressed with existing or forthcoming equipment enhancements. Some of the limitations stem from the decision to validate the concept with a minimal viable system, before committing additional engineering resources to address all the requirements of a fully operational system.

Current bioacoustic observing systems struggle to monitor remote, expansive oceanic habitats over extended periods (seasonal and interannual). The Sonophore addresses this limitation through its implementation as a profiling drifting buoy within the acoustic-based observation and modelling system proposed by Handegard et al.^27^. Operating autonomously for sustained periods, the Sonophore delivers essential data for biogeochemical and ecosystem model validation and parameterisation. Importantly, acoustic backscatter has recently been recognised as a Global Ocean Observing Systems (GOOS) Essential Ocean Variable and Essential Climate Variable for zooplankton and micronekton^28^. This underscores the growing international consensus on the value of acoustic observations for sustained ecosystem monitoring and highlights the relevance of platforms like the Sonophore in supporting coordinated, globally consistent ocean observing efforts.

## Conclusion

The integration of established technologies, such as autonomous profiling floats equipped with acoustic sensors, represents a future approach to monitoring the ocean’s largest living biomass reservoir. This methodology can deliver continuous and cost-effective observations of mesopelagic communities at ocean basin scale and can provide the data needed to improve both scientific understanding and practical management decisions in a time of rapid ocean change. Continued development and deployment of platforms like the Sonophore will be key to resolving large gaps in our understanding of ecological patterns in oceanic ecosystems and informing adaptive, climate-resilient management strategies.

## Methods

Here we instrument two autonomous profiling floats with two echosounders with different nominal frequencies to validate the proof of concept. We have termed this platform the *Sonophore*, derived from the Greek meaning of *sono* (sound) and *phoros* (to carry), as the buoyancy engine of the autonomous profiling float will effectively carry an *echosounder* vertically through the water column.

### Autonomous profiling float

The MRV-Alto, an autonomous profiling float, was selected due to its large buoyancy change capacity (800 cc). The general principle of a MRV float is that it inflates/deflates an external bladder with oil from an internal reservoir, to increase/decrease its volume without changing its mass, thereby decreasing/increasing its density and causing the float to rise/descend in the water column. Adding (echosounder) mass to a float displaces a greater quantity of seawater and requires a float with a relatively large buoyancy change capacity to profile. These floats were originally developed by Scripps Institution of Oceanography and are used in the Core Argo program, are depth rated to 2000 m, achieve over 500 dives in a lifetime and have an Iridium modem for telemetry, command and control. As adapted from Johnson and Fassbender^29^ the Argo program is an international, global array of profiling floats^30^ that has revolutionized observational physical oceanography and climate science^31^. Its public real-time data are contributed by many nations and used for a wide range of purposes, including weather forecasting. Regional pilot arrays that were initiated at the turn of the millennium grew to a sparse global array of about 900 floats in 2005 and on to ~ 3900 floats in 2023.

### Echosounders

Two Kongsberg Wideband Autonomous Transceiver (WBAT) echosounders were selected as they are: pressure rated to 1500 m depth, battery powered, record data internally, and can operate two broadband transducers simultaneously (one of which can be split beam) at 2 Hz, a rate suitable to observe individual organisms. Each Sonophore was fitted with two wideband transducers (Table 1), operating at 35–45 kHz and 100–155 kHz on one and at 55–90 and 185–255 kHz on the other. The purpose of using different transducers was to test noise performance and provide some frequency diversity, noting that the two higher frequencies are preferred for characterising smaller micronekton^32^. Data were collected and analysed down to 35 m from the transducer to maximise ping rate, resolve individual organisms and avoid volume backscattering environments where possible. The transducers projected downwards to match the orientation of echosounders in established bio-acoustic observing programs, in anticipation that data products from the Sonophore will aid in the interpretation of bioacoustic datasets from Ships of Opportunity and mooring programs^33^.

**Table 1:** Configuration of echosounders on MRV profiling floats 11,509 and 11,506.

Float serial number	WBAT serial number	Transducer	Pulse type	Frequency range	Beam configuration	Beamwidth	Pulse duration (us)	Ping rate (Hz)	Range (m)
11,509	287,971	ES70-18CD	FM	55–90	single	18	1024	2	35
11,509	287,971	ES200-7CD	FM	185–255	split	7	1024	2	35
11,506	287,945	ES38-18DK	FM	35–45	single	18	1024	2	35
11,506	287,945	ES120-18CDK	FM	100–155	split	18	1024	2	35

### Platform tilt

Platform tilt is an important aspect of the Sonophore design to ensure the angle at which acoustic waves are transmitted is accounted for in analysis, due to the sensitivity of an organism’s cross sectional backscattering strength to its orientation. A Lowell instruments MAT1 Orientation acceleration and temperature logger was used to measure platform tilt. The accelerometer and magnetometer data were sampled at 16 Hz and averaged every second for reporting.

### Field tests

After a series of wharf tests, the first open water dives were conducted between the 11 and 12 November 2024 offshore of Maria Island, Tasmania. Firstly 60 m tethered and untethered dives were completed close to Maria Island, followed by a 24-h deployment in 600 m water depth. A second deepwater calibration trial was also conducted between 13 and 14 March 2025 offshore of Maria Island > 1200 m water depth. A third multiday deployment was carried out from 4 to 8 April and a 24-hour calibration exercise on during 9 April offshore of Bicheno, Tasmania. The experimental protocols/permits required to conduct this work were consistent with CSIRO policy requirements.

### Acoustic data analysis

#### Numerical density of scatterers (Density m^3^)

Timestamped depth data were collected by the profiling float, and these were inserted into the heave field in the echosounder files to provide the echosounder depth to the analysis software. There was very little noise in the data, so no noise removal was done. Tracks from individual organisms were extracted using the tracking module of KORONA (Korneliussen et al., 2016), a part of the LSSS acoustic analysis software (Korneliussen et al., 2006). Tracking parameters were the default for KORONA except that the minimum organism target strength was lowered to − 80 dB (re 1 m^2^), some 10 dB above the background noise level in the data. The full track data were then exported for processing – from this the target strength (TS) at 120 kHz for each ping in the track was extracted and the linear mean of these taken for each track. The mean depth for each track was extracted and used to visualise organism volumetric density (number of organisms per unit volume) and mean target strength as a function of depth and time of day. This initial analysis was restricted to one echosounder channel at one frequency to reduce processing complexity for this proof of concept work. The 120 kHz channel was chosen because it had a wide beam angle, was a split-beam transducer, and the frequency was high enough to detect the smaller micronekton organisms.

#### Echosounder calibration

Echosounder calibration is required to ensure accurate system performance through time and varying environmental conditions. A shallow water acoustic calibration facility was used to calibrate both Sonophores prior to deployment in March and April 2025 using the standard reference sphere calibration procedure^34^. To complement surface calibrations, two deepwater calibrations to 500 m and 1000 m depth were attempted in March and April 2025 respectively, to investigate depth dependent effects on system performance. For each of these experiments, a 38.1 mm diameter tungsten carbide calibration sphere was suspended ~ 10 m below the transducers and a 350 g ballast weight ~ 3 m below the sphere. The combined mass of the sphere and ballast weight (780 g) was constrained by the requirement that the Sonophore still be able to carry out profiles if the sphere and ballast weight were lost during the deployment.

## Data Availability

The dataset generated during the current study are available in the CSIRO Data Access Portal, 10.25919/1gkd-6r28.
